# The Influence of Parental Behaviours and Socioeconomic Factors on Offspring’s Precursors of Atherosclerosis: A Systematic Review and Meta-Analysis of Observational Studies

**DOI:** 10.3390/ijerph22121791

**Published:** 2025-11-26

**Authors:** Ana Carvalhinho Silva, Filipe Maia, Francisco Estima, Anelise Gaya, Diego Christofaro, Jorge Mota, Luísa Aires, Gustavo Silva

**Affiliations:** 1 Research Center in Sports Sciences, Health Sciences and Human Development (CIDESD), University of Maia (UMaia), 4475-690 Maia, Portugal; fmaia.dcd@umaia.pt (F.M.); festima.dcd@umaia.pt (F.E.); 2 Graduate Program in Human Movement Sciences, Federal University of Rio Grande do Sul (PPGCMH-UFRGS), Porto Alegre 90690-200, RS, Brazil; anelise.gaya@ufpel.edu.br; 3 School of Physical Education and Physiotherapy, Federal University of Pelotas (ESEF-UFPEL), Pelotas 96055-630, RS, Brazil; 4 Graduate Program in Movement Sciences, Department of Physical Education, School of Technology and Sciences, São Paulo State University (PPGCM-FCT-UNESP), Presidente Prudente 19060-900, SP, Brazil; diego.christofaro@unesp.br; 5 Research Centre in Physical Activity, Health, and Leisure (CIAFEL), Faculty of Sport, University of Porto (FADE-UP), 4050-313 Porto, Portugal; jmota@fade.up.pt (J.M.); luisaaires.7566@esag-edu.net (L.A.); 6 Laboratory for Integrative and Translational Research in Population Health (ITR), 4050-091 Porto, Portugal

**Keywords:** arterial stiffness, blood pressure, socioeconomic status, educational level, children, adolescents, parents

## Abstract

Parents can influence both the behaviours and cardiovascular health of their school-aged children and adolescents. Understanding these effects is essential for developing targeted interventions. This systematic review and meta-analysis aims to report existing research findings on the associations between parental lifestyle behaviours, socioeconomic status (SES), and educational level (EDL) and offspring’s arterial stiffness (AS) and blood pressure (BP). Following PRISMA methodology, 19 observational studies were selected from the PubMed, Scopus, and EBSCO databases. Inclusion criteria focused on age range, children’s AS and BP, parental behaviours, SES, and EDL. The STROBE checklist ensured the quality and reliability of the included studies. The meta-analysis included data from three or more studies. Higher levels of maternal physical activity were associated with lower AS in offspring. Parental smoking was associated with increased AS and higher BP in offspring. The meta-analyses showed that a lower EDL was associated with higher AS in offspring, 0.22 (95% CI: 0.15, 0.29; *p* < 0.001). No significant associations were found between parental SES and offspring’s systolic (*p* = 0.40) and diastolic (*p* = 0.50) BP. Parental behaviours shape offspring’s cardiovascular health. Parental physical activity and higher EDL seem to be protective, while parental smoking is associated with adverse outcomes. Further research is needed to clarify these relationships.

## 1. Introduction

Early atherosclerotic changes and alterations in blood pressure (BP) in children and adolescents are increasingly recognised as critical determinants of lifelong cardiovascular risk. Atherosclerosis is a chronic, progressive process initiated by endothelial dysfunction and lipid accumulation, which gradually reduces arterial elasticity. These structural changes lead to increased arterial stiffness, thereby elevating blood pressure and cardiac workload [1,2,3,4]. Persistent exposure to adverse lifestyle behaviours accelerates these vascular alterations through oxidative stress, inflammation, and endothelial damage [1,5]. Although arterial stiffness is traditionally associated with ageing, emerging evidence also shows that such vascular alterations can begin in childhood and adolescence [6,7,8].

Modifiable risk factors contribute to the early onset and progression of atherosclerosis and elevated blood pressure, including excess adiposity, dyslipidaemia, insulin resistance, and low levels of physical activity [1,2,5,9]. These conditions promote endothelial dysfunction, oxidative stress, vascular remodelling, and increased arterial stiffness [1,2,5,9]. In contrast, regular physical activity and healthy lifestyle behaviours exert protective effects on vascular health by improving body mass index, body composition, endothelial function, and arterial elasticity, while reducing oxidative stress and inflammation [5,10,11,12,13,14]. Maintaining good sleep habits and reducing sedentary time are also associated with lower BP and improved cardiometabolic profiles. Replacing 30 min of sedentary time with an equivalent duration of sleep or physical activity has been linked to improvements in cardiovascular health, reductions in cardiometabolic risk factors [12], and increased physical fitness levels [15]. Furthermore, excessive screen time, including television and electronic devices, contributes to an increase in body adiposity and metabolic changes that accelerate atherosclerosis progression [12,16].

Family dynamics encompass specific characteristics, behaviours, and conditions that directly affect the development of cardiovascular disease (CVD). A comprehensive understanding of the family context, including family history, genetic predisposition, and shared behaviours, is crucial for the prevention and management of cardiovascular conditions. Although genetic factors establish a baseline susceptibility to CVD, modifiable lifestyle behaviours provide a means of transforming inherent risk into preventive benefit. This makes the study of family behaviours particularly relevant, as they hold the potential to improve health outcomes and mitigate the consequences of genetic predispositions [17,18,19,20]. Furthermore, the family environment plays a crucial role in shaping healthy behaviours in children and adolescents. Parents who encourage physical activity and sports and promote an active lifestyle create a favourable context for their children’s cardiovascular health. Studies indicate that parental participation in joint physical activity and regulating screen time can contribute to the prevention of hypertension and early endothelial dysfunction [17,21,22]. Parents play an essential role as role models for healthy behaviours and facilitators of practices promoting cardiovascular health. By encouraging sports participation, promoting regular physical activity, and providing transport to sports activities, parents help to strengthen healthy habits from childhood, forming a solid foundation for long-term CVD prevention [17,18,21,22,23]. In addition, socioeconomic status (SES) and parental educational levels (EDLs) influence children’s behaviours and cardiovascular health [24,25,26,27]. Studies indicate that children from lower-income families have higher rates of obesity, hypertension, and dyslipidaemia, due to limited access to sports facilities, activities, and environments that encourage an active lifestyle [22,28]. Parental education also affects adherence to healthy habits, as more educated parents tend to foster a more favourable environment for the prevention of chronic diseases [27,29,30]. Disparities in SES and EDL can lead to differences in health maintenance and disease prevention opportunities. Research suggests that SES and EDL affect cardiovascular health outcomes and the effectiveness of disease prevention strategies [31].

Despite growing evidence, important gaps remain in understanding the full extent of parental influence on offspring cardiovascular health. This study aims to provide new insights into how parental behaviours and socioeconomic factors influence the development of atherosclerosis in offspring, identifying key determinants that can inform targeted interventions and future preventive strategies, and highlighting the need for further research to develop effective interventions. Accordingly, this systematic review and meta-analysis summarises the existing evidence on the associations between parental lifestyle behaviours, socioeconomic status, and educational attainment and atherosclerosis and blood pressure in school-aged offspring, specifically focusing on children and adolescents.

## 2. Materials and Methods

### 2.1. Protocol and Registration

The systematic review was registered on the International Prospective Register of Systematic Reviews (PROSPERO) database (registration ID: CRD42024512141) and followed the Preferred Reporting Items for Systematic Reviews and Meta-Analyses (PRISMA) guidelines [32]. A completed PRISMA checklist is provided in the Supplementary Material (File S1).

### 2.2. Inclusion and Exclusion Criteria

The systematic review targeted studies involving school-aged children and adolescents aged 5 to 18 years, along with their respective parents, both healthy and with clinical conditions linked to lifestyle behaviours. Both observational and experimental studies were initially considered. However, due to the lack of experimental studies, only observational studies (cohort/longitudinal, case–control, and cross-sectional) focusing on atherosclerosis, blood pressure, parental lifestyle behaviours, and familial contexts for SES and EDL were included. Only English-language, peer-reviewed journal articles were considered for inclusion—grey literature, such as theses, dissertations, conference abstracts, and non-peer-reviewed reports, was excluded. The search included all studies available up to the date of consultation, with no restrictions on the starting publication year or follow-up duration.

Exclusion criteria included quasi-experimental studies, case reports, case series, opinion articles, letters to the editor, commentaries, conference proceedings, policy articles, reviews, meta-analyses, and study protocols without baseline data. Additionally, animal studies and those focusing on genetics, heritability, or behaviours during pregnancy were excluded.

### 2.3. Search Strategy

As observed in Table 1, the advanced search strategy employed the PICO method (Patient/Population, Intervention, Comparison, and Outcome): Patient/population—children and adolescents; Intervention—parental lifestyle; Comparison—several lifestyle factors such as PA, inactivity, sedentary behaviour, smoking, SES, and EDL; Outcomes—offspring’s arterial stiffness indicators and blood pressure.

The research was conducted on the following electronic databases: MEDLINE via PubMed, SCOPUS, and EBSCO (Academic Search Complete and SportDiscus). All databases were consulted on 31 March 2025. The search included all studies available up to that date, with no restriction on the starting publication year.

The search terms included controlled vocabulary (MeSH terms) and all possible entry terms. For instance, for “arterial stiffness”, both “arterial stiffness” and related terms were searched. The search queries combined key concepts and Boolean operators.

Thus, the PubMed query search was the following: (“parental lifestyle” [Text Word] OR “parents lifestyle” [Text Word] OR “parents physical activity” [Text Word] OR “parental physical activity” [Text Word] OR “parental exercise” [Text Word] OR “parental sleep” [Text Word] OR “parents exercise” [Text Word] OR “parents sleep” [Text Word] OR “mother-child relations” [MeSH Terms] OR “mother child relationship *” [Text Word] OR “parent–child relations” [Text Word] OR “parents” [MeSH Terms]) AND (“children” [Text Word] OR “young age” [Text Word] OR “child” [Text Word] OR “adolescents” [Text Word] OR “young people” [Text Word] OR “teenage” [Text Word] OR “youth” [Text Word] OR “youth *” [Text Word] OR “child” [MeSH Terms] OR “child *” [MeSH Terms] OR “youth *” [MeSH Terms] OR “adolescent” [MeSH Terms]) AND (((“arterial stiffness” [Text Word] OR “vascular stiffness” [Text Word] OR “aortic stiffness” [Text Word] OR “pulse wave velocity” [Text Word] OR “augmentation index” [Text Word] OR “central aortic pressure” [Text Word] OR “central blood pressure” [Text Word] OR “peripheral blood pressure” [Text Word] OR “pulse pressure” [Text Word] OR “atherosclerosis” [Text Word] OR “atherogenesis” [Text Word] OR “carotid intima-media thickness” [All Fields]) AND “vascular stiffness” [MeSH Terms]) OR “carotid artery diseases” [MeSH Terms] OR “blood pressure” [MeSH Terms] OR “pulse wave analysis” [MeSH Terms] OR “arterial pressure” [MeSH Terms] OR “atherosclerosis” [MeSH Terms]).

Equivalent searches were performed on the Scopus, EBSCO Academic Search Complete, and EBSCO SportDiscus databases, adjusting the query search to the specificities of each database (Appendix A, Table A1).

### 2.4. Study Selection, Data Extraction

**Figure 1:**
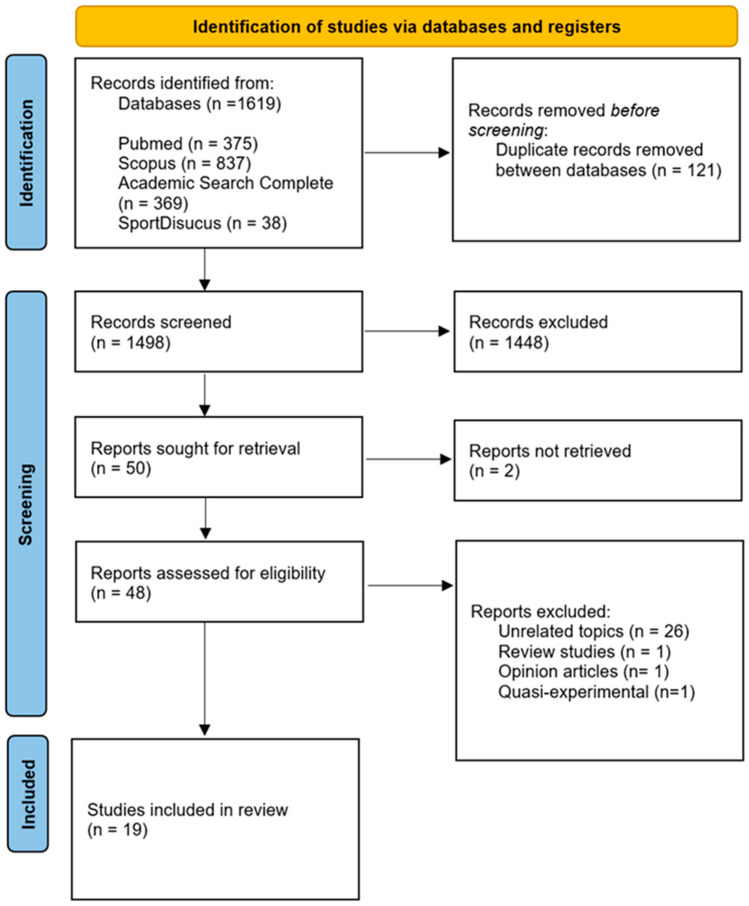
Flowchart diagram of screening studies included in the systematic review.

Figure 1 illustrates the study selection process. Data from the search was exported to Excel Software (Microsoft Corp.), and duplicates were removed. Articles were initially screened by title and abstract, followed by a full-text assessment under the inclusion and exclusion criteria presented in Section 2.2. Two investigators (ACS and FM) independently evaluated the articles, resolving discrepancies by discussing them with a third investigator (GS).

### 2.5. Quality Assessment of the Included Studies

The quality of studies was assessed using the STROBE checklist, ensuring the inclusion of essential items for observational studies (Table 2).

Each study was evaluated according to key sections and subsections. These included the title and abstract (1); introduction, including background/rationale (2) and objectives (3); methods, covering study design (4), setting (5), participants (6), variables (7), data sources/measurement (8), bias (9), study size (10), quantitative variables (11), statistical methods (12), participants (13), and descriptive data (14); outcome data (15), main results (16), and other analyses (17); discussion, including key results (18), limitations (19), interpretation (20), and generalisability (21); and other information such as funding (22). Each item was evaluated using the following coding: “Plus” (+) indicated that the study adequately met the criteria, “Less” (−) indicates partial or insufficient reporting, and “NA” refers to not applicable for the study.

Overall, the included studies demonstrated varying levels of adherence to reporting standards outlined by the STROBE scale. Although the study designs and objectives were often well-described, some studies lacked clarity in addressing potential biases and statistical methods. Common limitations were inadequate explanations of statistical methods, limited sensitivity analyses, and limited efforts to address potential biases. Some studies lacked detailed descriptions of handling missing data, which may have compromised the robustness of the results.

### 2.6. Data Analysis

All statistical analyses were performed using Review Manager (RevMan) version 5.4 (Cochrane Collaboration).

A random-effects meta-analysis was conducted using restricted maximum likelihood estimation (REML) to assess the effects of parental EDL on offspring’s PWV and the effects of SES on offspring’s BP. Standardised mean differences (SMDs) were calculated for each study to compare the offspring with lower vs. higher parental EDL in the PWV analysis, and lower vs. higher SES background in the BP analysis, with effect sizes (ESs) expressed as Hedges’ g to reduce potential bias from small sample sizes. Effect sizes were interpreted using the following thresholds: <0.20 as negligible, 0.20–0.49 as small, 0.50–0.79 as moderate, and >0.80 as large. Statistical significance was set at *p* < 0.05, and 95% confidence intervals (CI) were reported. Between-study heterogeneity was quantified using the I2 statistic, which indicated the portion of total variation due to heterogeneity rather than random error. Values of 25%, 50%, and 75% were interpreted as low, moderate, and high heterogeneity, respectively. Subgroup analyses were performed to explore potential sources of heterogeneity

## 3. Results

The PRISMA flowchart illustrates the screening details of the systematic review search (Figure 1). Out of 1619 initially identified records, 1498 were screened. From this, 50 full-text articles were sought for retrieval, of which 2 could not be retrieved. Ultimately, 48 reports were assessed for eligibility, and 19 observational studies met the predefined criteria for inclusion in this systematic review. These included fourteen studies on blood pressure, three on arterial stiffness, and two on both topics. Of the selected studies, thirteen were cross-sectional, collecting data at a single point in time, and six were cohort or longitudinal studies.

Additionally, 29 reports, comprising review articles, opinion pieces, or quasi-experimental designs, were excluded due to their lack of relevance to the study topic. This process ensured that only the most relevant observational studies were included in the review. The systematic review included only observational studies on arterial stiffness and blood pressure in school-aged children and adolescents, focusing on parental lifestyle behaviours, SES, and EDL. Only studies published in English on healthy children and adolescents, or those affected by lifestyle-related factors, were considered.

The included studies had sample sizes ranging from 94 to 3946 participants, ages ranging from six to twenty, across several countries. Due to the limited number of available studies, two meta-analyses were conducted, considering (a) parental SES and offspring systolic (SBP) and diastolic (DBP) blood pressure (four studies); (b) parental EDL and offspring arterial stiffness, using pulse wave velocity (PWV) as the outcome (three studies). Furthermore, no studies were found on sleep or sedentary behaviour despite its inclusion in the search criteria.

### 3.1. Systematic Review

#### 3.1.1. Arterial Stiffness

Table 3 presents studies on arterial stiffness in children and adolescents, focusing on parental physical activity and smoking habits. The study by Koechli et al. [45] reported a statistically significant association (*p* = 0.035) between higher levels of parental PA and lower PWV—measured to assess arterial stiffness in school-aged offspring—with a particular association observed in mothers (*p* = 0.040), while the association for fathers did not reach statistical significance (*p* = 0.068). After adjusting for parental smoking, the associations were attenuated and lost statistical significance (mother: *p* = 0.085; fathers: *p* = 0.124). Similarly, Lona and Hauser [33] found that maternal PA levels were associated with lower PWV in children (*p* = 0.049), while no significant association was observed for paternal PA (*p* = 0.483). The findings suggest that maternal PA has a residual influence on arterial stiffness at early offspring ages.

Moreover, parental smoking was consistently associated with worse cardiovascular outcomes in offspring. Specifically, Koechli et al. [45] reported significantly higher PWV values in children exposed to parental smoking (*p* < 0.001), with a stronger association observed for paternal smoking (*p* < 0.001) compared to maternal smoking, which did not reach significance (*p* = 0.219). In another study, Kallio et al. [46] observed no significant differences in flow-mediated dilatation (FMD) response at a single time point (*p* = 0.054), but longitudinal exposure to high cotinine levels was associated with significant changes in FMD response patterns, indicating impaired endothelial function in chronically exposed school-aged offspring.

The association between parental education level (EDL) and arterial stiffness, as shown in Table 4, in offspring, showed mixed results. Koechli et al. [45] found a significant inverse association between higher parental EDL and lower PWV in offspring (*p* = 0.041), but this association was not significant when adjusting for household income (*p* = 0.420). Additionally, when analysed separately by parent, the associations did not reach statistical significance (*p* > 0.05). Similarly, Lona et al. [49] and Bouthoorn et al. [25] found no significant association between parental EDL and offspring’s PWV.

Regarding parental socioeconomic status (SES), Koechli et al. [45] observed significantly lower PWV in offspring from high SES households compared to medium and low SES, both before (*p* = 0.002) and after adjusting for EDL (*p* = 0.033). However, Lona et al. [33] found no significant differences in PWV across SES groups. Studies in low- and middle-income countries also reported inconsistent findings: Balogun et al. [37], in Nigeria, found no significant association between SES and pulse pressure (*p* = 0.214), and Kinra et al. [44], in India, reported mixed results depending on sex and model adjustment. For boys, a significant association was found (*p* = 0.035), but after full adjustment, the association lost significance. 

#### 3.1.2. Blood Pressure

Table 5 provides an overview of the studies examining the relationship between children’s and adolescents’ blood pressure and parental physical activity. Hacke and Weisser [35] found that children with at least one physically active parent had lower exercise SBP (*p* = 0.013), while no significant association was found for resting SBP. Conversely, Khanolkar et al. [39] reported no significant association between parental PA and children’s SBP or DBP.

Considering smoking habits, as shown in Table 6, Simonetti et al. [34] reported an appositive association between school-aged offspring’s SBP and parental smoking habits, which intensified with the cumulative number of parent-related risk factors. Similarly, Zhang et al. [40] reported increased SBP in girls exposed to parental smoking, with these girls having 1.11 times higher odds for developing hypertension. However, no such association was observed in boys.

Table 7 and Table 8 report, respectively, the studies assessing blood pressure in offspring and parental educational level and SES. Hacke and Weisser [35] found that a low parental EDL was associated with higher exercise SBP, but not with resting SBP. Khanolkar et al. [39] reported that school-aged offspring from lower parental EDL groups had higher SBP compared to those from higher EDL groups (*p* < 0.005). Other studies similarly found associations between lower parental EDL and higher offspring SBP [34,41,47]. Bouthoorn et al. [25] reported an inverse association between maternal EDL and school-aged offspring’s SBP, while Kvaavik et al. [38] found that higher paternal EDL was associated with lower offspring SBP.

Khanolkar et al. [39] found that the children of skilled workers had higher SBP when compared to peers whose fathers had a higher occupational class. Kaczmarek et al. [47] found a higher prevalence of SBP hypertension among offspring living in rural areas and among children from economically inactive families or those experiencing income inadequacy. Similar SES-related associations with school-aged offspring’s SBP were reported in other studies from Nigeria and India [37,43,44]. However, Kinra et al. [44] reported that these associations with SBP were weak and inconsistent, disappearing after adjustments, while Ansa et al. [43] found the association only in girls.

Kwok et al. [41] and Calbano et al. [42] did not report a significant association. Leino et al. [48] reported that boys with fathers with a lower SES had significantly higher mean SBP compared to those with fathers in higher SES groups.

Regarding DBP, Kinra et al. [44] and Ansa et al. [43] showed significant associations between parental SES and school-aged offspring’s DBP, particularly in girls. Khanolkar et al. [39] reported no significant associations between parental PA and young age offspring’s DBP, whereas Kaczmarek et al. [47] reported a higher prevalence of DBP levels in rural areas and among children with parents who had lower incomes. Simonetti et al. [34] and Balogun et al. [37] found mixed results, with some SES indicators associated with DBP and others not.

### 3.2. Meta-Analyses

**Figure 2:**
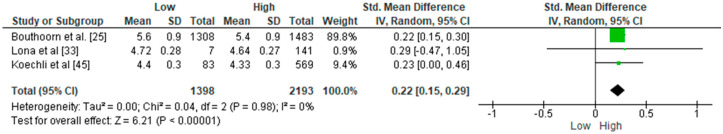
Meta-analysis of children’s pulse wave velocity and parents’ educational level. The green squares represent the effect estimates for each study, with the size of the square proportional to the weight of the study in the overall meta-analysis; the green dots represent the point estimates of the SMD for each study; the rhombuses represent the combined effect from all studies, with the width of the rhombus showing the 95% confidence interval of the overall effect estimate. Tau^2^, Chi^2^ and I^2^ represent standard heterogeneity statistics in meta-analysis [25,33,45].

The meta-analysis performed on associations between parental EDL and children’s and adolescents’ PWV, shown in Figure 2, illustrated a small and statistically significant association between parental EDL and the offspring’s PWV The overall standardised mean difference (SMD) of 0.22 (95% CI: 0.15, 0.29; *p* < 0.001) suggests that offspring from lower educational backgrounds exhibit higher PWV values, indicating increased arterial stiffness compared to their peers from higher educational backgrounds. The lack of heterogeneity suggests consistency in the findings. After applying the trim and fill method, the adjusted values remained unaltered.

**Figure 3:**
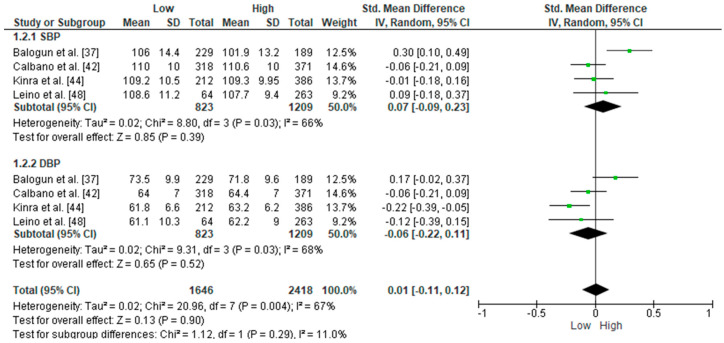
Meta-analysis of children’s blood pressure and socioeconomic status. The green squares represent the effect estimates for each study, with the size of the square proportional to the weight of the study in the overall meta-analysis; the green dots represent the point estimates of the SMD for each study; the rhombuses represent the combined effect from all studies, with the width of the rhombus showing the 95% confidence interval of the overall effect estimate. Tau^2^, Chi^2^ and I^2^ represent standard heterogeneity statistics in meta-analysis [37,42,44,48].

The meta-analysis of the associations between socioeconomic status and offspring’s blood pressure (Figure 3) reports no significant differences between high- and low-SES groups in systolic blood pressure and diastolic blood pressure overall. The effect sizes for SBP and DBP were not statistically significant between the high- and low-SES groups (*p* = 0.40 and *p* = 0.50, respectively). Heterogeneity was moderate for SBP (I^2^ = 66%) and moderate to high for DBP (I^2^ = 70%), indicating variability among the study results. The findings related to the offspring’s blood pressure were not statistically significant, and the trim and method did not alter the results.

## 4. Discussion

This systematic review and meta-analysis highlights the potential influence of parental behaviour and social context on cardiovascular outcomes in school-aged offspring, focusing on arterial stiffness and blood pressure. The meta-analysis highlights two main associations: (a) between parents’ educational level and the arterial stiffness of their children and adolescents’ offspring, measured by PWV, based on three studies; and (b) between parental socioeconomic status and offspring’s blood pressure, based on four studies. While the number of studies was limited and the quantitative meta-analysis was restricted to SES and EDL, the broader systematic review qualitatively explored additional parental behaviours beyond those included in the quantitative analysis, such as PA and smoking.

Considering parental PA and offspring’s cardiovascular health, evidence suggests a significant association between higher levels of maternal PA and lower PWV in school-aged offspring, suggesting a potential mother-driven protective association with offspring’s cardiovascular health [33,45]. In both studies, the association was observed in the maternal subgroup after adjusting for age and sex. In the study by Koechli et al. [45], a significant association was also reported when the analysis included both parents, whereas no significant results were reported for fathers alone. In contrast, Lona et al. [33] did not observe a significant association in the combined mothers and fathers group. Additionally, Koechli et al. [45] found that the significant associations were lost when the model was further adjusted for smoking status, suggesting that smoking may act as a confounding factor in the associations between parental PA and offspring PWV. Overall, these findings suggest a potential maternal-driven benefit of parental PA on the offspring’s atherosclerosis process, although more robust evidence is required. The number of studies included in this review focusing on parental PA and offspring’s atherosclerosis was limited to two. Both, however, employed similar methodologies and a similar age gap (6–8 years), assessing the same outcome (PWV), and using initial models adjusted for sex and age. The *p*-values for the significant results were borderline, with *p* = 0.035 [45] and *p* = 0.049 [33], suggesting marginal statistical significance. Both studies reported statistical power between 90% and 95%, suggesting that the borderline results were unlikely to be due to an inadequate sample size and may instead reflect modest effect sizes or the sensitivity of the associations to model adjustments. Despite the limited evidence base, the findings consistently suggested potential associations between higher levels of maternal PA and lower PWV values in school-aged offspring.

In the existing literature, physical activity has been widely studied in association with children and adolescents’ cardiovascular health, with evidence showing improvements in endothelial function, better indices of atherosclerosis, and reduced blood pressure levels [12,14,50,51,52,53]. Considering the limited number of studies included in this review, this represents a perspective on the influence of parents on their children’s and adolescents’ cardiovascular health. Furthermore, available evidence in the literature reports a positive association between parental physical activity levels and those of their offspring [54,55,56]. Combined with the well-established health benefits of physical activity in the literature [10,14,57], these findings suggest that parents may indirectly influence their children’s cardiovascular health by shaping their children’s physical activity habits.

This systematic review also identified two studies that reported significant associations between parental smoking habits and arterial stiffness in school-aged children and adolescents. Koechli et al. [45] reported significantly higher PWV values in offspring with smoking fathers, in both models adjusted for age and sex and for age, sex, and parental PA. Similarly, Kallio et al. [46] reported a significant reduction in the magnitude of the FMD response among children repeatedly exposed to high cotinine values. Moreover, the potential protective effect of parental PA identified in Koechli et al. [45] appeared to be attenuated or lost in the presence of parental smoking, suggesting that smoking may counteract or neutralise the indirect benefits of parental PA. Although these findings are consistent, the overall evidence remains sparse and limited, as it is drawn from a small number of studies with substantial methodological variability and varied outcomes, including PWV and FMD [45,46]. Regarding blood pressure outcomes, the evidence was more heterogeneous. Hacke and Weisser [35] reported a statistically significant increase in SBP during exercise in children exposed to parental smoking, although no differences were found at rest after adjustment for age, sex, height, BMI percentile, and physical fitness. Conversely, Li et al. [36] found that exposure to secondhand smoke was associated with a higher risk of higher SBP in offspring, which persisted after adjusting for potential confounders, although the effect size was moderate. Simonetti et al. [34] also reported a statistically significant association (*p* < 0.001), but with an R^2^ = 0.049, indicating that the model explained less than 1% of the variance, suggesting a very small effect despite its statistical significance. Another study by Zhang et al. [40] reported a significant positive association with school-aged girls across all three models applied (crude; adjusted for age, height, and BMI; and further adjusted for several lifestyle factors, maternal education level, and paternal hypertension), although no association was observed for boys. In contrast, two studies reported no significant associations [39,46]. For diastolic blood pressure, both Khanolkar et al. [39] and Zhang et al. [40] reported no significant associations. When analysing serum cotinine concentration categories (nondetectable, low, high), Zhang et al. [40] found no significant association. Simonetti et al. [34] reported a very small, but statistically significant, association in the univariate model that disappeared after full adjustment, suggesting confounding effects. Overall, mean DBP was slightly higher, approximately 3 mmHg, in children exposed to parental smoking compared with non-exposed peers, *p* = 0.01.

Evidence in adults shows that smoking leads to endothelial inflammation and contributes to higher blood pressure levels [58]. Similar negative associations were found in children and adolescents in the studies included in this review [45,46,59]. These findings indicate that, even at an early development stage, children and adolescents chronically exposed to secondhand tobacco smoke may already show early signs of compromised cardiovascular health. Such results underscore the complexity of lifestyle behaviours and highlight the need to consider both positive and negative parental influences simultaneously. Understanding these interactions within the broader family and environmental context is essential, as they collectively shape offspring’s cardiovascular outcomes.

The association between parental EDL and indices of arterial stiffness in children appears to be weak and inconsistent. Koechli et al. [45] reported a small but statistically significant difference in means between the low-, medium-, and high-education categories when the model was adjusted for age and sex (*p* = 0.042); however, this association was lost after further adjustment for household income. Both Lona et al. [33] and Bouthoorn et al. [25] did not find significant associations. Despite the weak strength and consistency of individual findings, the methodological homogeneity of the outcomes and comparability of samples allowed a meta-analysis, which revealed a statistically significant inverse association between parental EDL and school-aged offspring PWV (SMD = 0.22; 95% CI: 0.15–0.29; *p* < 0.001), with low heterogeneity. Although the effect size was small, this finding reinforces the relevance of parental education as a predictor of offspring’s early vascular changes. Considering SES, the evidence is more heterogeneous. One study found a significant inverse association between SES and children’s and adolescents’ PWV (*p* = 0.002; *p* = 0.033 after adjusting for smoking), as well as lower AIx values among boys from higher SES groups (*p* = 0.035) [40,47]. Others found no significant associations with offspring PWV. The meta-analysis did not detect significant differences in systolic or diastolic blood pressure between high- and low-SES groups (*p* = 0.40 and *p* = 0.50, respectively), with moderate to high heterogeneity (I^2^ = 66% for SBP; I^2^ = 70% for DBP). Although SES has been more extensively investigated [37,39,42,44,47], inconsistencies may stem from different measurement approaches, including parental income, composite indices, or occupation, making direct comparisons difficult.

Overall, the evidence linking EDL and SES to arterial stiffness in children and adolescents is limited and inconsistent, with variability in outcomes (e.g., PWP, AIx, and PP), SES indicators, and statistical approaches. These variables may also reflect broader social advantages and disadvantages rather than direct biological pathways. Parents with higher educational backgrounds are more likely to create a structured routine, encourage and promote a healthier balance between physical activity and sedentary behaviour in children [29], and limit exposure to harmful habits such as indoor smoking [60]. These factors collectively contribute to more favourable cardiovascular profiles in offspring, including lower PWV values and higher physical activity levels [45,61,62].

This systematic review and meta-analysis identifies a significant gap in studies exploring the associations between parental 24 h movement behaviours—particularly regarding parental sleep and sedentary behaviours—and their influence on offspring cardiovascular health. Advancing knowledge in this area is crucial for developing effective policies and interventions fitted to families’ needs. Parental behaviours and socioeconomic factors are particularly relevant, suggesting the need for targeted actions such as family-based education programmes or community-level health promotion strategies.

The primary limitations focus on the limited number of studies, low effect sizes, statistical inconsistency, and methodological heterogeneity. The predominance of cross-sectional designs limits the ability to infer causality, and most studies did not report power analyses or justify sample size, raising concerns about false-positive findings, overfitting, and statistical noise [63,64]. In several cases, associations lost significance after adjustment, weakening the conclusions. For example, Lona et al. [33] found a marginal association between maternal PA and offspring PWV, which was lost when the statistical analyses considered both parents. Similarly, in Koechli et al. [45], statistically significant results found from the initial model were lost in subsequent models after further adjustments. Challenges also arose from a lack of standardisation in both exposure and outcome assessments. Blood pressure was measured using different devices (e.g., Dinamap Compact T monitor, Tech Med TM-Z mercury gauge sphygmomanometer, and oscillometric devices), and arterial stiffness was assessed using several indices, including PWV, AIx, and PP, which vary in sensitivity and clinical relevance. Inconsistencies were also present in the classification and operationalisation of SES, a multidimensional concept encompassing income, education, occupation, and other contextual factors. Differences across studies may affect cardiovascular outcomes in distinct ways, likely contributing to heterogeneity and complicating interpretation. Additionally, this topic raises a key methodological challenge. Children and adolescents do not exhibit clinical manifestations or significant changes in traditional cardiovascular indicators. However, atherosclerosis and hypertension can begin developing early in life, highlighting the importance of establishing healthy lifestyle habits during childhood [1,2,6,7,8]. Subclinical markers such as PWV are sensitive to early changes in vascular function, and even minor alterations detected serve as early indicators of future cardiovascular risk, a concept supported by existing evidence [65,66,67].

In addition to these methodological constraints, some broader and self-critical considerations should be acknowledged. Although the search strategy was comprehensive and aligned with the aims of this review, it was restricted to three major databases and English-language, peer-reviewed publications, which introduces potential language and publication bias. The exclusion of grey literature and experimental evidence further constrains the generalisability and causal interpretation of the findings. Independent screening and data extraction were undertaken by multiple reviewers, although some degree of subjectivity in study selection cannot be entirely excluded.

Despite the application of rigorous inclusion and quality assessment criteria, the synthesis relied on studies employing heterogeneous definitions of socioeconomic status, educational level, and cardiovascular indicators, which may have led to residual confounding. The inclusion of studies with differing methodological quality in the meta-analysis, although necessary to achieve statistical power, may also have increased between-study heterogeneity. From a conceptual perspective, the review primarily focused on parental physical activity, sedentary behaviour, smoking, socioeconomic status, and educational level, while other potentially relevant lifestyle dimensions, such as diet, alcohol intake, psychological stress, or hereditary influences, were not considered.

Future investigations using longitudinal or experimental designs are needed to clarify causal mechanisms, apply standardised exposure and outcome measures, and empirically test the proposed ecological–behavioural pathways underlying early cardiovascular risk.

The novelty of this review lies in its integrative approach, which combines evidence on both parental behaviours and social contexts while considering early vascular health markers in school-aged children and adolescents. It underscores that cardiovascular risk begins within the microsystem of the family, where behavioural modelling and environmental exposures converge. These findings extend Bronfenbrenner and Morris’s ecological model [68], which conceptualises human development as occurring within multiple environmental layers. According to this framework, parents shape their children’s behaviour and health through the environment they create and the interactions they foster [69]. By focusing primarily on parental behaviours, this review highlights how parents may have a mediating or moderating role in influencing their school-aged offspring’s cardiovascular health.

Beyond its empirical synthesis, this review advances the theoretical understanding of how parental and contextual factors interact within the family microsystem to shape early cardiovascular risk. By linking parental educational level, socioeconomic status, and health behaviours to subclinical vascular markers, the findings empirically substantiate and refine Bronfenbrenner and Morris’s ecological model [68], illustrating how proximal processes, such as parental modelling, shared routines, and health-related patterns, mediate the influence of distal social structures like socioeconomic status. These results extend the ecological framework by demonstrating that subclinical cardiovascular indicators, including arterial stiffness and blood pressure, can serve as measurable outcomes within this model. This integration provides a more comprehensive view of how social and behavioural environments are biologically embedded across development. Furthermore, by highlighting the potential moderating roles of parental behaviours, the review complements social learning and intergenerational health transmission theories, suggesting that cardiovascular risk is not solely biologically inherited but also dynamically shaped through behavioural pathways. Collectively, these insights encourage future theoretical integration between ecological, behavioural, and physiological perspectives to explain how lifestyle contexts influence cardiovascular health from early life forward.

From a practical perspective, these findings provide valuable insights for designing family-based cardiovascular prevention strategies. Interventions that simultaneously promote parental PA, reduce or end parental smoking habits, and incorporate health education could generate synergetic benefits for both generations. Schools, community health programmes, and policymakers should recognise parents as key agents of behavioural modelling and include them in early prevention initiatives, emphasising the family unit as the main target of intervention rather than focusing only on children and adolescents. Moreover, the findings align with the United Nations Sustainable Development Goals (SDGs), particularly those related to promoting good health and well-being (SDG 3), reducing social disparities (SDG 10), and strengthening health, family, and community environments (SDG 11). Evidence in the literature shows that parental behaviours can be modified through family- and school-based interventions and programmes that promote physical activity, reduce sedentary time, and foster healthier home environments. Such preventive strategies should not only educate parents but also leverage the family context to promote lasting health habits. Effective programmes combine encouraging physical activity, reducing sedentary time, fostering healthier home environments, and engaging parents through meetings, assignments, or informational material [70,71]. By highlighting the early influence of parents, this review supports a primary prevention approach that begins in childhood, emphasising the importance of early monitoring of cardiovascular health.

Overall, this systematic review contributes novel evidence highlighting the multidimensional pathways through which parental education, socioeconomic context, and health-related behaviours collectively shape offspring cardiovascular health. This synthesis provides a valuable foundation for future longitudinal studies and intervention trials aimed at breaking the intergenerational cycle of cardiovascular risk.

## 5. Conclusions

This systematic review underscores a critical gap in the literature regarding parental behaviours and their specific associations with key movement behaviours—namely physical activity, sedentary behaviour, and sleep—as well as their impact on early markers of atherosclerosis in children and adolescents. The meta-analysis further reveals that lower parental educational attainment is significantly associated with increased atherosclerosis in offspring, as evidenced by higher pulse wave velocity. Conversely, no consistent associations were found between parental socioeconomic status and offspring blood pressure, despite isolated studies suggesting a potential influence. These findings highlight the need for further research to clarify the pathways through which parental behaviours and socioeconomic factors may influence early cardiovascular risk in youth.

## Figures and Tables

**Table 1:** Search strategy by the PICO method.

Patient/Population	Intervention	Comparison	Outcomes
Children and Adolescents	Parental Lifestyle Behaviours	Parental Lifestyle Behaviours Sleep Physical Activity Sedentary Behaviours Smoking SES EDL	Arterial stiffness: PWV, AIx, IMT, and PP Blood Pressure

SES = Socioeconomic status; EDL = Educational level; PWV = Pulse wave velocity; AIx = Augmentation Index; PP = Pulse pressure; IMT = Intima media thickness.

**Table 2:** Quality assessment of the included studies using the STROBE checklist of items that should be included in reports of observational studies.

	T/A		I		M														R											D				O
	1		2	3	4	5	6		7	8 *	9	10	11	12					13 *			14 *			15 *	16			17	18	19	20	21	22
	(a)	(b)					(a)	(b)						(a)	(b)	(c)	(d)	(e)	(a)	(b)	(c)	(a)	(b)	(c)		(a)	(b)	(c)						
Lona et al. [33]	+	+	+	+	+	+	+	NA	+	+	+	+	+	+	+	−	+	−	+	+	+	+	+	+	+	+	+	−	+	+	+	+	+	+
Simonetti et al. [34]	−	+	+	+	−	+	+	NA	+	+	+	−	+	+	+	−	+	−	+	+	−	+	−	NA	+	+	+	−	+	+	+	+	+	+
Hacke and Weisser [35]	+	+	+	+	+	+	+	NA	+	+	+	−	+	+	+	+	+	−	+	+	−	+	−	NA	+	+	+	−	+	+	+	+	+	+
Kwok et al. [30]	+	+	+	+	+	+	+	+	+	+	+	−	+	+	+	+	+	+	+	+	−	+	+	+	+	+	+	−	+	+	+	+	+	+
Li et al. [36]	+	+	+	+	+	+	+	NA	+	+	+	−	+	+	+	+	+	+	+	−	−	+	−	NA	+	+	+	−	+	+	+	+	+	+
Balogun et al. [37]	+	+	+	+	+	+	+	NA	+	+	+	−	+	+	+	−	+	−	−	−	−	+	−	NA	+	+	+	−	+	+	+	+	+	+
Bouthoorn et al. [25]	+	+	+	+	+	+	+	NA	+	+	+	−	+	+	+	+	+	−	−	−	−	+	+	NA	+	+	+	−	+	+	+	+	+	+
Kvaavik et al. [38]	+	+	+	+	+	+	+	+	+	+	+	−	+	+	+	+	+	−	+	−	−	+	+	+	+	+	+	−	+	+	+	+	+	+
Khanolkar et al. [39]	+	+	+	+	+	+	+	NA	+	+	+	−	+	+	+	−	+	−	+	−	−	+	−	NA	+	+	+	−	+	+	+	+	+	+
Zhang et al. [40]	+	+	+	+	+	+	+	NA	+	+	+	−	+	+	+	+	+	+	+	−	−	+	+	NA	+	+	+	−	+	+	+	+	+	+
Kwok et al. [41]	+	+	+	+	+	+	+	NA	+	+	+	−	+	+	+	+	+	−	+	+	−	+	+	NA	+	+	+	−	+	+	+	+	+	+
Calbano et al. [42]	+	+	+	+	+	+	+	NA	+	+	+	−	+	−	+	−	+	−	+	+	−	+	+	NA	+	+	+	−	−	+	+	+	+	+
Ansa et al. [43]	+	+	+	+	+	+	+	NA	+	+	+	−	+	+	−	−	+	−	+	−	−	+	+	NA	+	+	+	−	+	+	+	+	+	−
Kinra et al. [44]	+	+	+	+	+	+	+	NA	+	+	+	+	+	+	+	−	+	−	+	+	−	+	+	NA	+	+	+	−	−	+	+	+	+	+
Koechli et al. [45]	+	+	+	+	+	+	+	NA	+	+	+	+	+	+	+	−	−	+	+	+	+	+	+	NA	+	+	+	−	−	+	+	+	+	−
Schreier and Chen [24]	+	+	+	+	+	+	+	+	+	+	+	−	+	+	+	−	−	−	+	−	−	+	−	+	+	+	+	−	+	+	+	+	+	−
Kallio et al. [46]	+	+	+	+	+	+	+	+	+	+	+	−	+	+	+	−	−	+	+	−	−	+	−	+	+	+	+	−	+	+	+	+	+	+
Kaczmarek et al. [47]	+	+	+	+	+	+	+	NA	+	+	+	−	+	+	+	−	+	−	+	−	−	+	−	NA	+	+	+	−	+	+	+	+	+	+
Leino et al. [48]	+	+	+	+	+	+	+	NA	+	+	+	−	+	−	−	−	−	−	+	−	−	+	−	NA	+	+	+	−	−	+	−	+	+	−

T/A = Title and abstract; I = Introduction; M = Methods; R = Results; D = Discussion; O = Other Information; NA = Not applicable; + = item fulfilled; − = item not fulfilled; * Information reported separately for cases and controls in case-control studies and, if applicable, for exposed and unexposed groups in cohort and cross-sectional studies.

**Table 3:** Reports on the association between offspring’s arterial stiffness, parental physical activity, and smoking habits.

Authors (Year)	Sample Size	Age	Parents	Country	Method (Device)	Outcome	Model Adjustment	Main Results
Parental Physical Activity								
Koechli et al. [45]	833	6–8	Both	Switzerland	Oscillometric Mobil-OGraph	PWV	M1: age and sex M2: age, sex and smoke	Both Mean (95%CI): M1: L = 4.39 (4.35; 4.44); M = 4.32 (4.27; 4.37); H = 4.33 (4.30; 4.36); *p* = 0.035; M2: L = 4.38 (4.34; 4.43); M = 4.32 (4.28; 4.37); H = 4.34 (4.31; 4.36); *p* = 0.098 Mother Mean (95%CI) M1: L = 4.37 (4.34; 4.41); M = 4.31 (4.27; 4.35), H = 4.33 (4.30; 4.37); *p* = 0.040; Mother M2: L = 4.37 (4.33; 4.40); M = 4.31 (4.27; 4.35); H = 4.34 (4.30; 4.38); *p* = 0.085 Father M1 Mean (95%CI): L = 4.37 (4.34; 4.41); M = 4.32 (4.27; 4.36), H = 4.33 (4.30; 4.36); *p* = 0.068; Father M2: L = 4.37 (4.34; 4.40); M = 4.32 (4.27; 4.36), H = 4.33 (4.30; 4.36); *p* = 0.124
Lona et al. [33]	223	6–8	Both	Switzerland	Oscillometric Mobil-OGraph	PWV	Age and sex	Both Mean (95%CI): L = 4.73 (4.65; 4.82); M = 4.62 (4.57; 4.68); H = 4.66 (4.6; 4.72), *p* = 0.108 Mother Mean (95%CI): L = 4.7 (4.64; 4.77); M = 4.69 (4.62; 4.75); H = 4.6 (4.54; 4.66), *p* = 0.049 Father Mean (95%CI): L = 4.69 (4.62; 4.76); M = 4.64 (4.56; 4.71); H = 4.65 (4.6; 4.71), *p* = 0.483
Parental Smoking Habits								
Koechli et al. [45]	833	6–8	Both	Switzerland	Oscillometric Mobil-OGraph	PWV	M1: age and sex M2: age, sex, and parental PA	Both Mean (95%CI) M1: No = 4.32 (4.29; 4.34); Yes = 4.39 (4.35; 4.42), *p* < 0.001; M2: No = 4.32 (4.29; 4.34); Yes = 4.39 (4.35; 4.42), *p* = 0.001 Mother Mean (95%CI): M1: No = 4.33 (4.31; 4.36); Yes = 4.37 (4.32; 4.41); *p* = 0.219; M2: No = 4.33 (4.31; 4.36); Yes = 4.37 (4.32; 4.41); *p* = 0.247 Father M1: No = 4.33 (4.31; 4.36); Yes = 4.37 (4.32; 4.41), *p* < 0.001; M2: No = 4.32 (4.30; 4.34); Yes = 4.39 (4.36; 4.43), *p* = 0.001
Kallio et al. [46]	402	8–11	Both	Finland	Vascular Sonographer	FMD	Covariates with *p* < 0.10 in preliminary analyses were included in multivariable models using backwards stepwise selection.	A single measurement of cotinine effects over FMD responses in offspring at a single time point showed no statistical significance; *p* = 0.054. Offspring longitudinal exposure to high cotinine levels leads to significant changes in the FMD pattern’s response, time-by-group interactions (*p* < 0.001), and the magnitude of response (*p* = 0.002)

PWV = Pulse wave velocity; FMD = flow-mediated dilation, PA = Physical activity; M1 = Model adjustment 1; M2 = Model adjustment 2; L = Lown; M = Medium; H = High.

**Table 4:** Reports on the association between offspring’s arterial stiffness, parental educational level and socioeconomic status.

Authors (Year)	Sample Size	Age	Parents	Country	Method (Device)	Outcome	Model Adjustment	Main Results
Parental Educational Level								
Koechli et al. [45]	833	6–8	Both	Switzerland	Oscillometric Mobil-OGraph	PWV	M1: age and sex M2: age, sex, and HI	Both Mean (95%CI): M1 L = 4.40 (4.34; 4.47); M = 4.37 (4.33; 4.42); M2: H = 4.33 (4.30; 4.35), *p* = 0.041; L = 4.39 (4.31; 4.46); M = 4.33 (4.28; 4,38); H = 4.34 (4.31; 4.37), *p* = 0.420 Mother Mean (95%CI): M1: L = 4.39 (4.34; 4.44); M = 4.35 (4.31; 4.39); H = 4.33 (4.30; 4.35), *p* = 0.051; M2: L = 4.37 (4.32; 4.43); M = 4.33 (4.29; 4.37); H = 4.34 (4.31; 4.37), *p* = 0.386 Father Mean (95%CI): M1: L = 4.37 (4.32; 4.42); M = 4.37 (4.33; 4.41), H = 4.33 (4.30; 4.35); *p* = 0.118; M2: L = 4.35 (4.29; 4.41), M = 4.34 (4.29; 4.38), H = 4.34 (4.31; 4.37), *p* = 0.918
Lona et al. [33]	223	6–8	Both	Switzerland	Oscillometric Mobil-OGraph	PWV	M3: age, sex, and EDL M4: age, sex, and HI	M3: L = 4.72 (4.52; 4.93); M = 4.68 (4.62; 4.75); H = 4.64 (4.6; 4.69), *p* = 0.544 M4: L = 4.77 (4.54; 5); M = 4.68 (4.61; 4.75); H = 4.64 (4.59; 4.69), *p* = 0.466
Bouthoorn et al. [25]		6	Mother	Netherlands	Automatic Complior Device	PWV	Sex, age, and ethnicity	L = 0.06 (−0.02; 0.14); ML = 0.02 (−0.05; 0.09); MH = 0.03 (−0.05; 0.11), H = Reference; *p* = 0.22
Parental Socioeconomic Status								
Koechli et al. [45]	833	6–8	Both	Switzerland	Oscillometric Mobil-OGraph	PWV	M1: age and sex M2: age, sex, and EDL	Mean (95%CI) M1: L = 4.37 (4.33; 4.41); M = 4.37 (4.33; 4.41); H = 4.29 (4.26; 4.33), *p* = 0.002; M2: L = 4.36 (4.32; 4.41); M = 4.37 (4.34; 4.41); H = 4.30 (4.26; 4.34); *p* = 0.033
Lona et al. [33]	223	6–8	Both	Switzerland	Oscillometric Mobil-OGraph	PWV	M2: age and sex M1: age, sex, and EDL	Mean (95%CI) M1: L = 4.56 (4.43; 4.68), M = 4.66 (4.57; 4.75), H = 4.69 (4.58; 4.79), *p* = 0.273; Both M2: L = 4.6 (4.51; 4.69); M = 4.67 (4.6; 4.73); H = 4.68 (4.62; 4.74), *p* = 0.382
Balogun et al. [37]	229	8–20	Both	Nigeria	Computation PP = SBP-DBP	PP	Age, height, and weight	Mean ± SD: L = 31 ± 12.5; M = 32.2 ± 12.7, H = 30.3 ± 12.1; *p* = 0.2141 (Fratio 1.54)
Kinra et al. [44]	862	13–18	Both	India	Sphygmocor Apparatus	AIx	M1: age, sex, NS, AR and HR M2: M1 + Height M3: M2 + PS, FMI and cPSR	Mean ± SD Girls: L = 6.8 (12.3); M = 3.9 (11.1); H = 4.8 (9.9), *p* = 0.365; Mean ± SD Boys: L = 5.1 (10.4); M = 3.8 (9.8); H = 1.8 (10.9), *p* = 0.035 B (95%CI) M1: −1.21 (−2.36; −0.06), *p* = 0.04; B (95%CI) M2: −0.72 (−1.84; 0.40), *p* = 0.20; B (95%CI) M3: −0.63 (−1.72; 0.45), *p* = 0.24

PWV = Pulse wave velocity; PP = Pulse pressure; AIx = Augmentation index; M1 = Model adjustment 1; M2 = Model adjustment 2; M3 = Model adjustment 3; M4 = Model adjustment 4; EDL = Educational level; HI = Household income; NS = Nutritional supplementation; AR = Ambient room; HR = Heart rate; PS = Pubertal stage; FMI = Fat max index; cPSR = Central peripheral skinfold ratio; L = Low; M = Medium; ML = Mid-Low; MH = Mid-High; H = High.

**Table 5:** Reports on the association between offspring’s blood pressure and parental physical activity.

Authors (Year)	Sample Size	Age	Parents	Country	Method (Device)	Outcome	Model Adjustment	Main Results
Hacke and Weisser [35]	532	12–17	Both	Germany	Standard Stethoscope and Sphygmomanometer	SBP (rest) SBP (exercise)	Age, sex, height, BMI percentile, and physical fitness	Physical Inactivity Mean ± SB Rest:1.0 (0.1; 1.9); *p* = 0.467 Mean ± SB Exercise = 4.99 (3.5; 6.4), *p* = 0.013
Khanolkar et al. [39]	1204	5–14	Both	Swedish	Dinamap Compact T’Monitor	SBP	Age; gender, PS, family clustering, and parental EDL, occupation, and height	Total MET (hours/week) B (95%CI) Mother: ≤20.25 = Reference Group; Group 20.26–33.75 = −0.91 (−2.68; 0.85); Group ≥33.76 = −0.52 (−2.36; 1.30) Father: ≤7.5 = Reference Group; Group 7.6–25.5 = −1.26 (−3.15; 0.62); Group ≥25.6 = −0.69 (−2,48; 1.10) Total hours of vigorous PA/week (min/week) B (95%CI) Mother: None = Reference Group; Group ≤75 = 0.06 (−3.64; 3.78); Group 76–150 = −1.18 (−4.93; 2.56); Group >150 = −0.21 (−4.13; 3.70) Father: None or ≤30 = Reference Group; Group 60 = −0.74 (−3.92; 2.44); Group 90–120 = −0.76 (−4.00; 2.45); Group 150–360 = −0.06 (−3.09; 2.96); Group >360 = −3.72 (−7.94; 0.51)
Khanolkar et al. [39]	1171	5–14	Both	Swedish	Dinamap Compact T’Monitor	DBP	Age; gender, PS, family clustering, and parental EDL, occupation, and height	Total MET (hours/week) B (95%CI) Mother: ≤20.25 = Reference Group; Group 20.26–33.75 = 0.15 (−0.87; 1.18); Group ≥33.76 = −0.40 (−1.42; 0.63) Father: ≤7.5 = Reference Group; Group 7.6–25.5 = −0.48 (−1.61; 0.65); Group ≥25.6 = −0.17 (−1.28; 0.93) Total hours of vigorous PA/week (min/week) Mother: None = Reference Group; Group ≤75 = −0.82 (−2.97; 1.32); Group 76–150 = −0.96 (−3.13; 1.20); Group >150 = −1.36 (−3.60; 0.88) Father: None or 30 = Reference Group; Group 60 = 0.77 (−0.92; 2.46); Group 90–120 = 1.47 (−0.25; 3.21)

SBP = Systolic blood pressure; DBP = Diastolic blood pressure, BMI = Body Mass Index; PS = Pubertal Stage; EDL = Educational Level.

**Table 6:** Reports on the association between offspring’s blood pressure and parental smoking habits.

Authors (Year)	Sample Size	Age	Parents	Country	Method (Device)	Outcome	Model Adjustment	Main Results
Hacke and Weisser [35]	532	12–17	Both	Germany	Standard Stethoscope and Sphygmomanometer	SBP (rest) SBP (exercise)	Age, sex, height, BMI percentile, and physical fitness	Mean ± SB Rest = 0.1 (−0.7; 0.5), *p* = 0.945 Mean ± SB Exercise = 4.0 (3.1; 4.9), *p* = 0.030
Khanolkar et al. [39]	1171	5–14	Both	Swedish	Dinamap Compact T’monitor	SBP	Age; gender, PS, family clustering, and parental EDL, occupation, and height	Mother: N = Reference; F = −0.44 (−2.21; 1.32); C = −0.04 (−2.34; 2.33). Father N = Reference; F = 0.70 (−1.06; 2.50); C = −0.40 (−3.22; 2.40)
Simonetti et al. [34]	4236	5.7 ± 0.4	Both	Germany	Auscultatory Aneroid Sphygmomanometry	SBP	Full adjustment for potential confounders by multivariable Regression analysis identified	Univariate Regression B (SE): 0.0044 (0.0010); *p* < 0.0001, R^2^ = 0.0049
Kallio et al. [46]	402	8–11	Both	Finland	Standard Sphygmomanometer	SBP	Values are mean ± SD unless	Mean ± SB N = 104 ± 8; L = 104 ± 9; H = 104 ± 7; *p* = 0.99
Zhang et al. [40]	42.745	7–18	Both	China	Auscultation Mercury Sphygmomanometer	SBP Hypertension	Crude M1: Age, height, and BMI M2: several life factors; maternal EDL; parental hypertension	Girls B (95%CI): Crude = 1.03 (0.72; 1.34); *p* < 0.001; M1 = 0.6 (0.41; 0.96), *p* < 0.001; M2 = 0.44 (0.16; 0.72), *p* = 0.0002 Boys B (95%CI): Crude = 0.30 (−0.03; 0.64), *p* = 0.01 M1 = 0.24 (−0.04; 0.52), *p* = 0.09; M2 = 0.06 (−0.22; 0.33), *p* = 0.69 Hypertension OR (95%CI) Girls: Crude = 1.19 (1.10; 1.29), *p* < 0.001; M1 = 1.15 (1.06; 1.25), *p* < 0.001; M2 = 1.11 (1.02; 1.20, *p* = 0.01 Boys: Crude = 1.01 (0.93,1.09), *p* = 0.90; M1 = 0.96 (0.89; 1.04), *p* = 0.32; M2 = 0.93 (0.86; 1.01), *p* = 0.09
Li et al. [36]	3150	6–18	Both	China	standardised protocol	SBP BP	M1: age, sex, Tanner stage M2: M1 + residence, diet scores, parents’ EDL, parents’ BMI, and family history (diabetes)	Mean ± SB No-exposed = 106 ± 14; Parental smoking = 109 ± 14 M1 OR (95% CI) = 1.33 (1.13–1.58), *p* = 0.001 M2 OR (95% CI) = 1.22 (1.02–1.46), *p* = 0.028
Khanolkar et al. [39]	1171	5–14	Both	Swedish	Dinamap Compact T’Monitor	DBP	Age; height, gender, PS, family clustering, parental EDL, and occupation	Mother B (95%CI): Never = Reference; Former = 0.72 (−1.71; 0.28); Current = −0.46 (−1.75; 0.84). Father B (95%CI): Never = Reference; Former = 0.24 (−0.77; 1.26); Current = −0.62 (−1.92; 0.68)
Simonetti et al. [34]	4236	5.7 ± 0.4	Both	Germany	Auscultatory Aneroid Sphygmomanometer	DBP	Full adjustment for potential confounders by multivariable Regression analysis identified	Univariate Regression B (SE): 0.0030 (0.0012); *p* = 0.01, R^2^ = 0.0013 Full Model B (SE): 0.1608 (0.2686), *p* = 0.5.
Kallio et al. [46]	402	8–11	Both	Finland	Standard Sphygmomanometer	DBP	Values are mean_SD unless otherwise stated	Serum Cotinine Concentration Mean ± SB Nondetectable = 63 ± 5; Low = 64 ± 6; High = 63 ± 5; *p* = 0.98
Zhang et al. [40]	42.745	7–18	Both	China	Auscultation Mercury Sphygmomanometer	DBP Hypertension	Crude M1: Age, height, BMI M2: Several life factors; maternal EDL and parental hypertension	Girls B (95%CI): Crude = 0.60 (038; 0.83); *p* < 0.001; M1: 0.40 (0.19; 0.62); *p* < 0.001; M2: 0.26 (0.04; 0.47); *p* = 0.02 Boys B (95%CI): Crude = 0.18 (−0.06; 0.41), *p* = 0.14; M1: 0.13 (−0.08; 0.35); *p* = 0.23; M2: 0.0001 (−0.21; 0.21); *p* = 0.99
Li et al. [36]	3150	6–18	Both	China	standardised protocol	DBP	M1: Age, sex, Tanner stage M2: M1 + residence, diet scores, parents’ EDL, parents’ BMI, and family history (diabetes)	Mean ± SB No-exposed = 66 ± 10; Parental smoking = 69 ± 10, *p* < 0.001

SBP = Systolic blood pressure; DBP = Diastolic blood pressure, BP = Blood pressure; BMI = Body mass index; PS = Pubertal stage; EDL = Educational level.

**Table 7:** Reports on the association between offspring’s blood pressure and parental educational level.

Authors (Year)	Sample Size	Age	Parents	Country	Method (Device)	Outcome	Model Adjustment	Main Results
Schreier and Chen [24]	88	9–18	Both	Canada	BPM−100 automated BP monitor	SBP	Unadjusted Associations	12-month SBP for: Aggregate SBP B (SE) = −0.010 (0.243), *p* = 0.97 Trajectories of SBP B (SE) = 0.185 (0.187)
Hacke and Weisser [35]	532	12–17	Both	Germany	Standard Stethoscope And Sphygmomanometer	SBP (rest) SBP (exercise)	Age, sex, height, BMI percentile, and physical fitness	Rest MD (95%CI): 1.3 (0.6; 1.9), *p* = 0.315 Exercise MD (95%C): 5.2 (4.3; 6.2), *p* = 0.006
Khanolkar et al. [39]	1171	5–14	Both	Swedish	Dinamap Compact T’Monitor	SBP	Child’s age; gender, PS, family clustering, and parental EDL, occupation, and height	Mother B (95%CI): Other = 2.18 (−0.05; 4.42); Secondary = 2.17 * (0.63; 3.70); University = Reference; *p* < 0.05 * Father B (95%CI): Other = 2.72 * (0.11; 5.33); Secondary = 1.11 (−0.42; 2.65); University = Reference; *p* = *p* < 0.05 *
Calbano et al. [42]	1486	8–14	Mother	Argentina	Oscillometric Sphygmomanometer	SBP	Values are mean ± SD unless	Average SBP: Low = 110.24; Middle = 111.35; High = 110.68; *p* = 0.16
Bouthoorn et al. [25]	5843	6 (0.5)	Mother	Netherlands	Automatic Complior	SBP	Sex, age, and ethnicity	B (95%CI) Low = 2.28 * (1.62; 2.94); Mid-Low = 1.29 * (0.71; 1,87); Mid-High = 0.62 (−0.001; 1.24); High = Reference group; *p* < 0.001 *
Kvaavik et al. [38]	325	11–16	Both	Norway	1979 and 1981: a random-zero sphygmomanometer; 1991: dinamap 2006: instrument not recorded	SBP	Sex; intervention and maturation	Mother B (95%CI) = −0.10 (−0.21; 0.002) Father B (95%CI) = −0.14 (−0.24; −0.03)
Simonetti et al. [34]	4236	5.7 ± 0.4	Both	Germany	Auscultatory Aneroid Sphygmomanometry	SBP	Full adjustment for potential confounders by multivariable Regression analysis identified	Univariate regression B (SE) = −0.0053 (0.0009); *p* < 0.0010; R^2^ = 0.0068. Multiple Regression (Full Model) B (SE) = −0.5756 (0.3008), *p* = 0.06
Kwok et al. [41]	5604	~13	Both	Hong Kong	Oscillometric Device	SBP Prehypertension Hypertension	Unadjusted associations	Both SBP Mean (95%CI): Grade 9 or below = Reference, Grade 10–11 = −0.04 (−0.11; 0.02) Grade 12 or above = −0.11 * (−0.19; −0.04); Prehypertension in OR Grade 9 or below = 1.00; Grade 10–11 = 0.97 (0.84, 1.12); Grade 12 or above = 0.86 (0.73, 1.02); Hypertension in OR grade 9 or below = 1.00; Grade 10–11 = 0.84 (0.67; 1.05); Grade 12 or above = 0.78 (0.60; 1.02) Mother SBP Mean (95%CI): Grade 9 or below = Reference; Grade 10–11 = −0.08 * (−0.13; −0.02); Grade 12 or above = −0.11 * (−0.19; −0.03); Prehypertension in OR Grade 9 or below = 1.00; Grade 10–11 = 0.91 (0.79,1.04); Grade 12 or above = 0.92 (0.76,1.11); Hypertension in OR Grade 9 or below = 1.00; Grade 10–11 = 0.90 (0.73; 1.12); Grade 12 or above = 0.89 (0.66; 1.21) Father: SBP in mean difference Grade 9 or below = Reference; Grade 10–11 = −0.03 (−0.09; 0.04); Grade 12 or above = −0.10 * (−0.17; −0.03); Prehypertension in OR Below = 1.00; Grade 10–11 = 0.99 (0.86; 1.14); Grade 12 or above = 0.90 (0.76; 1.05); hypertension in OR Grade 9 or below = 1.00; Grade 10–11 = 1.00 (0.81; 1.25); Grade 12 or above = 0.82 (0.63, 1.07)
Kaczmarek et al. [47]	4941	10–18	Both	Poland	TECH MED TM-Z Mercury Gauge Sphygmomanometer	Prehypertension SBP Hypertension SBP	Multivariate analyses: FHH, sex, age, and weight status	Parents’ years of study Univariate analysis: Mother (<12 yrs) = 7.2%; (12 yrs) = 5.3%; (>12 yrs) = 3.5%; *p* = 0.002; Father: (<12 yrs) = 7.0%; (12 yrs) = 4.5%; (>12 yrs) = 2.7, *p* < 0.001 Results for hypertensive Maternal EDL Multivariate level (stepwise with backward elimination) in OR: Prehypertension (<12 yrs) = 1; (12 yrs) = 0.75 (0.69; 0.92); (>12 yrs) = 0.60 (0.48; 0.91); *p* = 0.002; hypertension (<12 yrs) = 1; (12 yrs) = 0.73 (0.62; 0.86); (>12 yrs) = 0.54 (0.39; 0.72), *p* = 0.0002
Khanolkar et al. [39]	1171	5–14	Both	Swedish	Dinamap Compact T’Monitor	DBP	Child’s age, gender, pubertal stage, family clustering, and parental EDL, occupation, and height	Mother B (95%CI): Other = 0.88 (−0.42; 2.20); Secondary = 1.37 * (0.47 2.27); *p* < 0.05 *; University = Reference Father B (95%CI): Other = 0.46 (−0.92; 1.42); Secondary = 0.51 (−0.40; 1.42); University = Reference
Calbano et al. [42]	1486	8–14	Mother	Argentina	Oscillometric Sphygmomanometer	DBP	Values are mean ± SD unless otherwise stated	Average DBP Low = 63.78; Middle = 65.66; High = 64.29; *p* = 0.04.
Bouthoorn et al. [25]	5843	6 (0.5)	Mother	Netherlands	Automatic Complior	DBP	Sex, age, and ethnicity	B (95%CI) Low = 1.80 (1.25; 2.35); *p* < 0.001; Mid-Low:1.27 (0.79; 1.76); *p* < 0.001; Mid-High = 0.69 (0.18; 1.21); *p* < 0.01; High = Reference
Kvaavik et al. [38]	325	11–16	Both	Norway	1979 and 1981: a random-zero sphygmomanometer; 1991: dinamap 2006: instrument not recorded	DP	Sex, intervention, and maturation	Mother: B (95%CI) = −0.03 (−0.14; 0.08) Father: B (95%CI) = −0.03 (−0.13; 0.08)
Simonetti et al. [34]	4236	5.7 ± 0.4	Both	Germany	Auscultatory Aneroid Sphygmomanometry	DBP	Full adjustment for potential confounders by multivariable Regression analysis identified	Univariate Regression B (SD) = 0.0048 (0.0011), *p* < 0.000; R^2^ = 0.0036 Multiple Regression (Full Model) B (SD) = −0.1851 (0.2619), *p* = 0.5
Kwok et al. [41]	5604	~13	Both	Hong Kong	Oscillometric Device	DBP	Unadjusted associations	Both B (95% CI): Low = Reference; Middle = −0.05 * (−0.08; 0.01); High = −0.07 * (−0.11; −0.04); Mother B (95% CI): Low = Reference; Mid = −0.06 * (−0.009; −0.02); High = −0.07 * (−0.11; −0.03) Father B (95% CI): Low = Reference; Middle = −0.04 * (−0.07; −0.003); High = −0.06 (−0.09; −0.02)
Kaczmarek et al. [47]	4941	10–18	Both	Poland	TECH MED TM-Z Mercury Gauge Sphygmomanometer	DBP	FHH, sex, age, and weight status	Univariate analysis: Mother Low = 6.1%; Middle = 3.6%; High = 2.5%; *p* < 0.001; Father: Low = 5.8%; Middle = 2.7%; High = 1.7, *p* < 0.001 Maternal EDL Multivariate level (stepwise with backward elimination) in OR: Prehypertension Low = 1; Middle = 0.66 (0.54; 0.81); High = 0.44 (0.29; 0.66); *p* < 0.0001; hypertension Low = 1; Middle = 0.60 (0.49; 0.74); High = 0.36 (0.24; 0.55), *p* < 0.0001

SBP = Systolic blood pressure; DBP = Diastolic blood pressure, BP = Blood pressure; BMI = Body mass index; PS = Pubertal stage; EDL = Educational level, FHH = Family history of hypertension; * Values statistically significant at p < 0.05.

**Table 8:** Reports on the association between offspring’s blood pressure and parental socioeconomic status.

Authors (Year)	Sample Size	Age	Parents	Country	Method (Device)	Outcome	Model Adjustment	Main Results
Khanolkar et al. [39]	1171	5–14	Both	Swedish	Dinamap Compact T’Monitor	SBP	Child’s age, gender, PS, family clustering, and parental EDL, occupation, and height	Occupation Mother B (95%CI): Higher = Reference; Lower = 0.43 (−1.78; 2.65); Skilled = 1.68 (−0.62; 3.98); Unskilled = 0.50 (−1.30; 2.27); Farmer = 2.28 * (−0.53; 5.10), *p* < 0.05 * Father B (95%CI): Higher = Reference; Lower = 1.68 (−0.61; 3.98); Skilled = 3.04 * (1.05; 5.03); Unskilled = 1.96 (−0.38; 4.30); Farmer = 0.92 (−1.85; 3.70), *p* < 0.05 *;
Calbano et al. [42]	1486	8–14	Mother	Argentina	Oscillometric Sphygmomanometer	SBP	Values are mean ± SD unless otherwise stated	Average SBP: Low = 110.06; Middle = 110.74; High = 110.66, *p* = 0. 074.
Kwok et al. [30]	4989	~13	Both	Hong Kong	Oscillometric Device	SBP Prehypertension Hypertension	Total effect, direct effect of parental education (effect not via mediators was adjusted for maternal and paternal ages and birthplace).	Absolute Income, *p* = 0.35 Indirect Effect (IE) B (95%CI) = 0.005 (−0.01; 0.020), Direct Effect € B (95%CI) = 0.09 (0.02; 0.16), Total Effect (TE) B (95%CI) = 0.10 (0.03; 0.16); Proportion Mediator (PM) B (95%CI) = 0.05 (−0.17; 0.35) Relative Income, *p* = 0.99 IE B (95%CI) = 0.01 (−0.03; 0.05); DE B (95%CI) = 0.08 (0.002; 0.16); TE B (95%CI) = (0.02; 0.16); PM B (95%CI) = (−0.41; 0.86)
Kaczmarek et al. [47]	4941	10–18	Both	Poland	TECH MED TM-Z Mercury Gauge Sphygmomanometer	Prehypertension SBP Hypertension SBP	FHH, sex, age, and weight status	Place of residence: A higher prevalence tendency of SBP hypertension in the rural areas group (9.0%, *p* < 0.001). Urban ≥ 100.000 inhabitants’ group is less likely to develop prehypertension OR = 0.56 (0.37; 0.82); *p* = 0.004 and hypertension OR = 0.40 (0.29; 0.55), *p* < 0.0001 than rural areas group Occupation: A higher prevalence tendency of SBP hypertension in the Economically inactive (EI) group (5.7%, *p* = 0.005 for fathers and 5.9%, *p* = 0.004 for mothers). This group is more likely to develop. Prehypertension OR = 1.53 (1.04; 2.25); *p* = 0.029 *p* < 0.0001 than those group of economically active Income Adequacy: higher prevalence of SBP hypertensive income inadequacy group (5.3%, *p* = 0.047. This is more likely to develop prehypertension OR = 1.40 (1.17; 1.94); *p* = 0.019 than those from income adequacy group
Balogun et al. [37]	229	8–20	Both	Nigeria	Electronic BP Monitoring Kit	SBP	Age, height, and weight	Income and Occupation Mean ± SD: Low = 106.0 ± 14.4; Middle = 05.0 ± 13.9; Upper = 101.9 ± 13.2; *p* = 0.0268, F ratio = 3.6
Ansa et al. [43]	964	10–17	Both	Nigeria	Accuson^®^ Mercury Sphygmomanometer	SBP	Values are mean ± SD unless otherwise stated	School’s Average Fees Girls Mean ± SD: Low5 = 106.8 ± 11.8; Low4 = 103.9 ± 11.3; Middle Class3 = 104.4 ± 12.3; Middle class2 = 105.7 ± 13.2; Upper = 100.8 ± 12.3; *p* = 0.009 Boys Mean ± SD: Low 5 = 07.4 ± 12.8; Low 4 = 108.5 ± 14.5; Middle Class 3 = 106.2 ± 12.4; Middle class 2 = 105.4 ± 11.4; Upper = 102.9 ± 12.3; *p* = 0.143
Kinra et al. [44]	862	13–18	Both	India	Oscillometric Device	SBP	M1: Age, sex, nutritional supplementation, AR, and HR M2: M1 + height M3: M2 + Pubertal stage, FTM, and cPSR	Standard of Living Index Mean ± SD Girls: Low = 107.5 (8.3); Middle = 107.5 (9.3); High = 107.2 (9.2); *p* = 0.917 Mean ± SD Boys: Low = 109.7 (11.1); Middle = 110.2 (10.7); High = 112.2 (10.9); *p* = 0.080 B (95%CI) M1: 0.55 (−0.23; 1.33); *p* = 0.16 B (95%CI) M2: 0.09 (−0.61; 0.78); *p* = 0.80 B (95%CI) M3: 0.15 (−0.58; 0.87); *p* = 0.68
Leino et al. [48]	1211	9, 12 and 15	Father	Finns	Zero Sphygmomanometer	SBP	Values are mean ± SD unless otherwise stated	Occupation Mean ± SD Girls: Upper level = 109.1 (9.1); F = 108.2 (10.5), *p* = 0.59 Mean ± SD Boys: Upper level = 106.3 (9.7); Boys F = 111.2 (12.3), *p* = 0.02
Khanolkar et al. [39]	1171	5–14	Both	Swedish	Dinamap Compact T’Monitor	DBP	Child’s age; gender, PS, family clustering, and parental EDL, occupation, and height	Mother B (95%CI): H = Reference; L = −0.27 (−1.64; 1.08); S = 0.45 (−0.75; 1.65); U = 0.20 (−0.88; 1.30); F = 1.24 (−0.73; 3.21) Father B (95%CI): H = Reference; L = 0.06 (−1.50; 1.63); S = 0.97 (−0.10; 2.05); U = 0.06 (−1.15; 1.30); F = 0.45 (−1.70; 2.60)
Calbano et al. [42]	1486	8–14	Mother	Argentina	Oscillometric Sphygmomanometer	DBP	Values are mean ± SD unless otherwise stated	Average DBP: Low = 64.03; Middle = 64.21; High = 64.44; *p* = 0.089
Kwok et al. [30]	4989	~13	Both	Hong Kong	Oscillometric Device	DBP Prehypertension Hypertension	Total effect, direct effect of parental education (effect not via mediators was adjusted for parents’ ages and birthplace).	Absolute Income, *p* = 0.10 Indirect Effect (IE) B (95%CI) = −0.001 (−0.01; 0.01); Direct Effect (DE) B (95%CI) = 0.04 (0.002; 0.08); Total Effect (TE) B (95%CI) = 0.04 (0.01; 0.08); Proportion Mediated (PM) B (95%CI) = −0.02 (−0.52; 0.42) Relative Income, *p* = 0.20 IE B (95%CI) = 0.02 (−0.005; 0.05); DE B (95%CI) = 0.02 (−0.02; 0.07); TE B (95%CI) = 0.04 (−0.005; 0.09); PM B (95%CI) = 0.38 (−1.07; 2.23)
Kaczmarek et al. [47]	4941	10–18	Both	Poland	TECH MED TM-Z Mercury Gauge Sphygmomanometer	Prehypertension DBP Hypertension DBP	FHH, sex, age, and weight	Place of Residence: A higher prevalence of DBP hypertension in the rural areas group (7.2%, *p* < 0.001). UH group is less likely to develop DBP prehypertension OR = 0.26 (0.17; 0.39); *p* < 0.0001 and hypertension OR = 0.17 (0.11; 0.27), *p* < 0.0001 than RA group Occupation: A higher prevalence tendency of DBP hypertension in fathers’ EI group (5.3%, *p* = 0.003) and mothers’ other group (7.0%, *p* < 0.001). Income Adequacy: A higher prevalence of DBP hypertensive in enough adequacy (5.5%, *p* = 0.041).
Balogun et al. [37]	229	8–20	Both	Nigeria	Electronic BP Monitoring Kit	DBP	Age, height, and weight	Income and Occupation Mean ± SD: Low = 73.5 ± 9.9; Middle = 72.9 ± 9.3; Upper = 71.8 ± 9.6; *p* = 0.1918, F ratio = 1.7
Ansa et al. [43]	964	10–17	Both	Nigeria	Accuson^®^ Mercury Sphygmomanometer	DBP	Values are mean ± SD unless otherwise stated	School’s Average Fees Girls Mean ± SD: Low5 = 68.5 ± 8.0; Low4 = 66.5 ± 9.1; Middle class3 = 64.6 ± 9.8; Middle class2 = 65.5 ± 9.8; Upper = 62.8 ± 9.8; *p* = 0.002 Boys Mean ± SD: Low5 = 18.1 ± 10.4; Low4 = 66.2 ± 9.7; Middle class3 = 65.4 ± 8.8; Middle class2 = 64.9 ± 9.5; Upper = 64.3 ± 9.3, *p* = 0.125 In girls, DBP was significantly associated with SES. The lower the SES, the lower the DBP
Kinra et al. [44]	862	13–18	Both	India	Oscillometric Device	DBP	M1: Age, sex, nutritional supplementation, AR, and HR M2: M1 + height M3: M2 + Pubertal stage, FMI, and cPSR	Standard of Living Index Girls: Low = 63.0 (5.7); Middle = 62.6 (7.0); High = 62.7 (6.0); *p* = 0.924 Boys: Low = 61.4 (6.8); Middle = 61.7 (6.5); High = 63.8 (6.4); *p* = 0.001 B (95%CI) M1: 0.54 (0.007; 1.00), *p* = 00.03; B (95%CI) M2: 0.40 (−0.07; 0.87); *p* = 0.09; B (95%CI) M3: 0.38 (−0.08; 0.84); *p* = 0.10
Leino et al. [48]	1211	9, 12 and 15	Father	Finns	Zero Sphygmomanometer	DBP	Values are mean ± SD unless otherwise stated	Mean ± SD Girls: I = 63.2 (9.6); F = 61.9 (10.0); *p* = 0.78 Mean ± SD boys: I = 61.1 (8.3); F = 58 (9.2); *p* = 0.09

SBP = Systolic blood pressure; DBP = Diastolic blood pressure, PS = Pubertal stage; EDL = Educational level, FHH = Family history of hypertension, FMI = Fat max index; cPSR = Central peripheral skinfold; * Values statistically significant at *p* < 0.05.

## Data Availability

The data sets generated during the current review are available from the corresponding author upon reasonable request.

## Supplementary Materials

The following supporting information can be downloaded at: https://www.mdpi.com/article/10.3390/ijerph22121791/s1, File S1: PRISMA 2020 checklist [32].

## Abbreviations

The following abbreviations are used in this manuscript:

AIx	Augmentation Index
AS	Arterial Stiffness
BP	Blood Pressure
CVD	Cardiovascular Disease
DBP	Diastolic Blood Pressure
EDL	Educational Level
FMD	Flow-mediated Dilation
IMT	Intima Media Thickness
PA	Physical Activity
PP	Pulse Pressure
PWV	Pulse Wave Velocity
SBP	Systolic Blood Pressure
SES	Socioeconomic Status
SMD	Standardised Mean Difference

## Appendix A

**Table A1:** Keywords used for Pubmed, Scopus, EBSCO (Academic Search Complete, and SportDiscus.

Search	Query
Pubmed	375
(“parental lifestyle” [Text Word] OR “parents lifestyle” [Text Word] OR “parents physical activity” [Text Word] OR “parental physical activity” [Text Word] OR “parental exercise” [Text Word] OR “parental sleep” [Text Word] OR “parents exercise” [Text Word] OR “parents sleep” [Text Word] OR “mother-child relations” [MeSH Terms] OR “mother child relationship *” [Text Word] OR “parent-child relations” [Text Word] OR “parents” [MeSH Terms]) AND (“children” [Text Word] OR “young age” [Text Word] OR “child” [Text Word] OR “adolescents” [Text Word] OR “young people” [Text Word] OR “teenage” [Text Word] OR “youth” [Text Word] OR “youth *” [Text Word] OR “child” [MeSH Terms] OR “child *” [MeSH Terms] OR “youth *” [MeSH Terms] OR “adolescent” [MeSH Terms]) AND (((“arterial stiffness” [Text Word] OR “vascular stiffness” [Text Word] OR “aortic stiffness” [Text Word] OR “pulse wave velocity” [Text Word] OR “augmentation index” [Text Word] OR “central aortic pressure” [Text Word] OR “central blood pressure” [Text Word] OR “peripheral blood pressure” [Text Word] OR “pulse pressure” [Text Word] OR “atherosclerosis” [Text Word] OR “atherogenesis” [Text Word] OR “carotid intima-media thickness” [All Fields]) AND “vascular stiffness” [MeSH Terms]) OR “carotid artery diseases” [MeSH Terms] OR “blood pressure” [MeSH Terms] OR “pulse wave analysis” [MeSH Terms] OR “arterial pressure” [MeSH Terms] OR “atherosclerosis” [MeSH Terms])	
Scopus	837
(“parental lifestyle” OR “parental exercise” OR “parental physical activity” OR “parents physical activity” OR “parents exercise” OR “parental behavior” OR “parental behavior” OR “parental sleep” OR “parents sleep” OR “parental sedentary behavior” OR “child parent relation” OR “child parent relation” OR “correlation, parent child” OR “correlation, parent child”) AND (children OR “young age” OR child OR adolescents OR “young people” OR young OR teenage OR youth * OR child * OR adolescent *) AND (“arterial stiffness” OR “vascular stiffness” OR “aortic stiffness” OR “aorta stiffness” OR “carotid intima-media thickness” OR “pulse pressure” OR “pulse wave velocity” OR “augmentation index” OR “central aortic pressure” OR “aortic pressure” OR “central blood pressure” OR “peripheral blood pressure” OR “carotid artery disease” OR atherosclerosis OR “atherosclerotic disease” OR “carotid artery diseases”)	
Academic Search Complete	369
(“parental lifestyle” OR “parental exercise” OR “parents exercise” OR “parental physical activity” OR “parents physical activity” OR “parents exercise” OR “parental behavior” OR “parental behavior” OR “parental sleep” OR “parents sleep” OR “parental sedentary behavior” OR mother OR father OR parents) AND (children OR “young age” OR child OR adolescents OR infant OR “young people” OR young OR teenage OR youth * OR child * OR adolescent *) AND (“arterial stiffness” OR “vascular stiffness” OR “aortic stiffness” OR “pulse pressure” OR “pulse wave velocity” OR “augmentation index” OR “central aortic pressure” OR “central blood pressure” OR “peripheral blood pressure” OR “carotid artery diseases” OR “carotid artery diseases” OR “carotid intima-media thickness”)	
SportDiscus	38
(“parental lifestyle” OR “parental exercise” OR “parents exercise” OR “parental physical activity” OR “parents physical activity” OR “parents exercise” OR “parental behavior” OR “parental behavior” OR “parental sleep” OR “parents sleep” OR “parental sedentary behavior” OR mother OR father OR parents) AND (children OR “young age” OR child OR adolescents OR infant OR “young people” OR young OR teenage OR youth * OR child * OR adolescent *) AND (“arterial stiffness” OR “vascular stiffness” OR “aortic stiffness” OR “pulse pressure” OR “pulse wave velocity” OR “augmentation index” OR “central aortic pressure” OR “central blood pressure” OR “peripheral blood pressure” OR “carotid artery diseases” OR “carotid artery diseases” OR “carotid intima-media thickness”)	

* Used as a truncation symbol to include word variations
